# Vagus nerve stimulation for upper limb motor impairment after ischemic stroke: A meta-analysis

**DOI:** 10.1097/MD.0000000000027871

**Published:** 2021-11-19

**Authors:** Yu-lei Xie, Shan Wang, Qing Wu, Xin Chen

**Affiliations:** Rehabilitation Medicine Department, Affiliated Hospital of North Sichuan Medical College, Nanchong, Sichuan, People's Republic of China.

**Keywords:** ischemic stroke, meta-analysis, randomized controlled trial, upper limb motor impairment, vagus nerve stimulation

## Abstract

**Background:**

Upper limb motor impairment is a common complication following stroke. Although few treatments are used to enhance motor function, still approximately 60% of survivors are left with upper limb motor impairment. Several studies have investigated vagus nerve stimulation (VNS) as a potential technique for upper limb function. However, the efficacy and safety of VNS on upper limb motor function after ischemic stroke have not been systematically evaluated. Therefore, a meta-analysis based on randomized controlled trial will be conducted to determine the efficacy and safety of VNS on upper limb motor function after ischemic stroke.

**Method:**

We searched PUBMED, MEDLINE, EMBASE, Cochrane Library, Web of Science, China National Knowledge Infrastructure Library (CNKI), and Wan Fang Database until April 1, 2021.

**Results:**

Six studies consisting of 234 patients were included in the analysis. Compared with control group, VNS improved upper limb function via Fugl-Meyer Assessment-Upper Extremity (mean difference = 3.26, 95% confidence interval [CI] [2.79, 3.74], *P* < .00001) and Functional Independence Measurement (mean difference = 6.59, 95%CI [5.77, 7.41], *P* < .00001), but showed no significant change on Wolf motor function test (standardized mean difference = 0.31, 95%CI [–0.15, 0.77], *P* = .19). The number of adverse events were not significantly different between the studied groups (risk ratio = 1.05, 95%CI [0.85, 1.31], *P* = .64).

**Conclusion:**

VNS resulted in improvement of motor function in patients after ischemic stroke, especially in the sub-chronic stage. Moreover, compared with implanted VNS, transcutaneous VNS exhibited greater efficacy in poststroke patients. Based on this meta-analysis, VNS could be a feasible and safe therapy for upper limb motor impairment.

## 1 Introduction

Stroke is a primary cause of mortality and associated morbidity worldwide.^[1]^ Approximately 60% of survivors after stroke suffer from upper limb motor impairment, which consecutively lead to loss of independence with poor quality of life.^[2,3]^ Therefore, it is essential to identify novel treatments for stroke survivors. Vagus nerve stimulation (VNS) either implanted or transcutaneous, is a neuromodulation therapy, which sends impulses into the neural center to generate corresponding nervous activity by stimulating the cervical vagus nerve.^[4,5]^ VNS has been widely applied to the clinical treatment of many diseases such as epilepsy, drug-refractory depression, pain, chronic tinnitus, and so on.^[6–10]^ Furthermore, VNS gradually shows a positive effect for the treatment of motor impairment after the stroke.^[11–13]^

Although the specific mechanism of VNS is not fully understood, studies have shown that VNS may activate the nucleus basalis neuron and locus coeruleus neuron, resulting in the widespread release of acetylcholine and norepinephrine in the cerebral cortex, respectively. The release of neurotransmitters eventually enhances the synaptic plasticity and the reorganization of cortical networks which ultimately improves motor function.^[14,15]^ Several randomized controlled trials (RCTs) both on animals and human have shown that VNS paired with rehabilitation training can be a potential option in terms of efficacy and safety on upper limb motor impairment after ischemic stroke.^[16–19]^ However, Dawson et al^[20]^ reported no significant change in motor function after VNS in the intention to treat analysis. Besides, a meta-analysis^[21]^ investigated the efficacy of VNS as the rehabilitation following stroke, which revealed a significant effect of VNS on Fugl-Meyer Assessment-Upper Extremity (FMA-UE). However, the conclusion was based on 3 RCTs with a small sample size with mixed models of ischemic and hemorrhagic stroke. Recently, some new researches evaluating the effect and safety of VNS on the motor function of ischemic stroke has emerged.

This meta-analysis aims to evaluate the efficacy and tolerability of VNS for upper limb motor impairment after ischemic stroke based on RCTs and attempted to provide clinical evidence for the VNS in the treatment of upper limb motor impairment after ischemic stroke.

## 2 Methods

This systematic review protocol was performed in accordance with the Preferred Reporting Items for Systematic Reviews and Meta-analysis Protocol (PRISMA-P). This is a literature based study, so ethical approval is not necessary.

### 2.1 Study search strategy

The methodology of this meta-analysis was done as recommended by the Cochrane Collaboration.^[22]^ The databases such as PUBMED, MEDLINE, EMBASE, Cochrane Library, Web of Science, China National Knowledge Infrastructure Library (CNKI), and Wan Fang Database were searched from inception until April 1, 2021, with the following keywords: vagus nerve stimulation and stroke. There were no restrictions on the language, region, race, or publication types.

### 2.2 Selection criteria

Patients diagnosed with ischemic stroke; Only RCTs comparing VNS paired with rehabilitation training and with only rehabilitation training; Studies having available completed valid data.

### 2.3 Data extraction and outcome measures

All of data were extracted independently by the 2 examiners, any disputes were settled by the consensus. In case of incomplete data, authors were contacted for details. For crossover trials, we only took the data for the first period (before crossover) into consideration.

The primary outcome included FMA-UE and the adverse events related to the therapy or devices, evaluating the efficacy and safety of VNS for upper limb impairment, respectively. The secondary outcomes included the Wolf motor function test (WMFT) and Functional Independence Measurement (FIM).

### 2.4 Quantitative and statistical analysis

All statistical analysis was performed by Review Manager 5.3 (The Cochrane Collaboration, Copenhagen, Denmark). Two independent examiners evaluated the quality of each RCT to estimate the risk of bias with the Cochrane risk of bias tool including sequence generation, allocation concealment, masking, incomplete outcome data, selective reporting, and other issues.^[23]^ We also utilized risk ratio to assess dichotomous outcomes and calculated 95% confidence intervals (CIs). Besides, mean difference (MD) and standardized mean difference (SMD) with 95%CI were assessed for continuous variables.

Heterogeneity in data of the selected study was assessed using the χ^2^ test and the I^2^ statistics. When I^2^ was less than 50% with a *P* value more than .1, there was no heterogeneity and therefore a fixed-effect model was used. On the contrary, if there was heterogeneity, we used a random-effect model to test the robustness of the results for the possible explanations. Furthermore, sensitivity and subgroup analysis was performed to find out the source of heterogeneity. However, due to the small number of included studies (n = 6), the publication biases could not be assessed.

## 3 Results

### 3.1 Study inclusion

**Figure 1:**
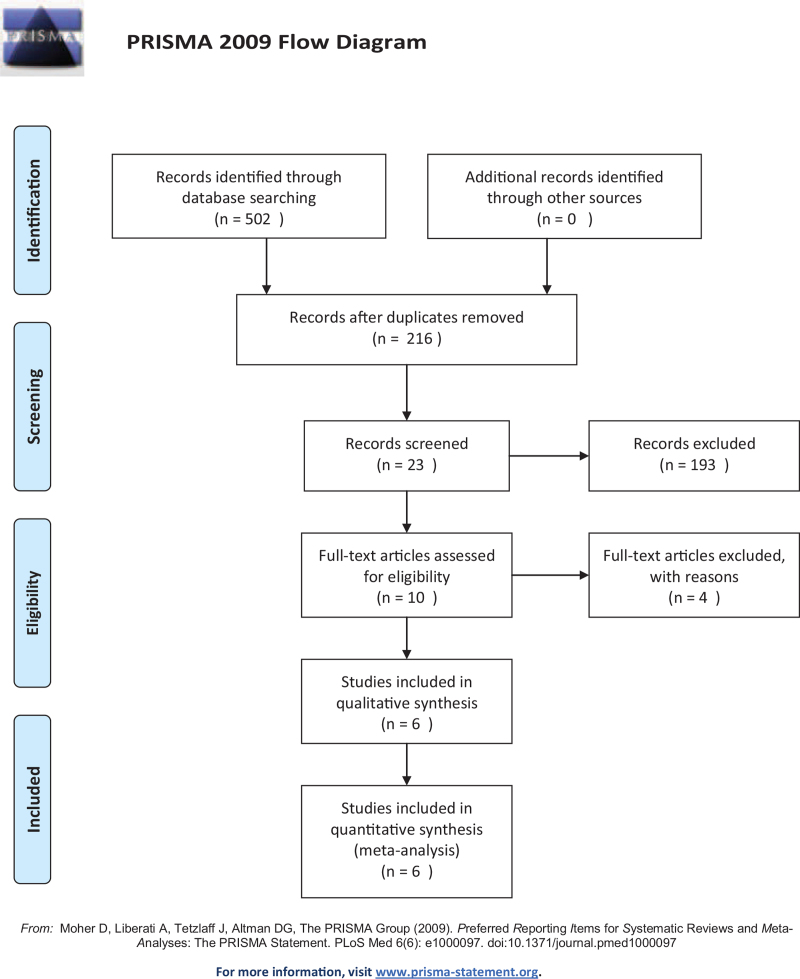
The PRISMA flow chart of the study selection process.

Figure 1 shows the flow chart of PRISMA. For the total of 502 studies identified by the predefined search strategy, 216 studies were selected after excluding the 286 duplications. Failing to meet the inclusion criteria, 193 studies were excluded through screening the abstracts and titles. Of the remaining 23 studies, 10 were sorted out after reading through the full text. One RCT was excluded for participants with both ischemic and hemorrhagic stroke,^[19]^ eventually, 6 studies were included in the analysis.^[17,20,24–27]^

### 3.2 Study characteristics

The characteristics of included studies are described in Table 1. A total of 234 patients were included in this meta-analysis. The sample size in the included studies, varied from 17 to 108. In each study, patients were randomly assigned to 2 groups: VNS paired with upper limb rehabilitation and upper limb rehabilitation alone. For 3 studies of implanted VNS, only 1 did not perform VNS device implantation in rehabilitation-only participants.^[20]^ For 3 studies of transcutaneous VNS, electrodes were fitted to the cymba conchae of the left ear, the sham group without electrical stimulation. While the stroke durations ranged from 1 month to years, the intervention lasted from 15 days to 6 weeks. Although more males than females were enrolled in the included studies, groups seemed balanced from sex. There was a 5-point significant difference (VNS group 40.10 ± 9.70 versus control group 45.30 ± 8.40) in the baseline of FMA-UE in the study of Dawson et al.^[20]^ Three studies^[25–27]^ employed transcutaneous VNS whereas 3 studies^[17,20,24]^ adopted implanted VNS as intervention. The stimulation parameters of VNS were different each study, such as stimulation intensity (mA), frequency (Hz), pulse width (μs), and duration (ms). Three studies^[17,20,24]^ employed the same stimulation settings of 0.8 mA, 30 Hz frequency, 100 μs pulse width with pulse train of 0.5 seconds. The measurements of effect mainly included FMA-UE, with other parameters such as WMFT, FIM, Brunnstrom stage, Ashworth, Box and Block Test, Nine-Hole Peg Test, and so on. The number of adverse events related to devices or therapy was chosen to evaluate safety of the employed VNS.

**Table 1:** Characteristics of included studies in the meta-analysis.

Study	Design	Patients		Duration of stroke		Gender (m/f)		Age(yr)		FMA-UE at baseline		Methods		Device parameters	Outcome measures		Adverse events
		Real	Sham	Real	Sham	Real	Sham	Real	Sham	Real	Sham	Real	Sham		Effect	Safety	
Wu, 2020	A single-blinded, RCT	10	11	36.30 ± 9.23 (d)	35.55 ± 6.47 (d)	5/5	8/3	64.50 ± 9.97	61.82 ± 10.63	17.50 ± 4.91	16.82 ± 3.89	Transcutaneous VNS plus rehabilitation training for 15 days	Sham VNS plus rehabilitation training for 15 days	Optimum intensity, 20 Hz, 300 μs, lasting 30 s per time of every 5 minutes, total of 1600 pulses	FMA-U,WMFT,FIM, Brunnstrom stage	HR; BP	Skin redness
Dawson, 2016	A blinded, open, RCT	9	11	1.8 ± 1.0 (y)	1.7 ± 1.3 (y)	7/2	9/2	57.9 ± 17.2	60.7 ± 10.7	40.1 ± 9.7	45.3 ± 8.4	Left VNS plus rehabilitation training for 6 weeks (18 times)	Only rehabilitation training for 6 weeks (18 times)	0.8 mA, 30 Hz, 100 μs, duration of 0.5 s	FMA-UE, ARA T, grip, and pinch strength	The number of serious adverse events related to therapy	Left vocal cord palsy and dysphagia; nausea; taste disturbance; hoarseness; neck tingling
Kimberley, 2018	A fully blinded, RCT	8	9	18 ± 0.5 (m)	18 ± 11.68 (m)	4/4	5/4	59.5 ± 7.4	60 ± 13.5	29.5 ± 6.4	36.4 ± 9.4	VNS plus rehabilitation training for 6 weeks (18 times)	Sham VNS plus rehabilitation training for 6 weeks (18 times)	0.8 mA, 30 Hz, 100 μs, duration of 0.5 s	FMA-UE, WMFT, Box and Block Test, Nine-Hole Peg Test, Stroke Impact Scale, and Motor Activity Log	The number of serious adverse events related to the device or therapy	Implantation wound infection; shortness of breath and dysphagia; hoarseness
Dawson, 2021	Triple-blinded, RCT	53	55	3.1 ± 2.3 (y)	3.3 ± 2.6 (y)	34/19	36/19	59.1 ± 10.2	61.1 ± 9.2	34.4 ± 8.2	35.7 ± 7.8	VNS plus rehabilitation training for 6 weeks (18 times)	Sham VNS plus rehabilitation training for 6 weeks (18 times)	0.8 mA, 30 Hz, 100 μs, duration of 0.5 s	FMA-UE, WMFT,MAL, SIS, SS-QOL, EQ-5D, BDI	NA	Vocal cord palsy
Wei, 2020	RCT	13	13	48.77 ± 24.74 (d)	50.38 ± 22.07 (d)	4/9	3/10	61.31 ± 11.54	57.23 ± 10.17	32.85 ± 12.13	28.31 ± 13.55	Transcutaneous left auricular VNS plus rehabilitation training for 4 weeks	Sham VNS plus rehabilitation training for 4 weeks	Optimum intensity, 25 Hz, 100 μs, lasting 30 s per time of every 30s	FMA-UE, Brunnstrom stage, MFAS, Ashworth	Electrocardiogram	Mild nausea and vomiting; mild pain in the left ear
Zhang, 2020	A triple-blinded, RCT	21	21	38 ± 1 5(d)	36.86 ± 2(d)	11/10	8/13	66.1 ± 1.49	64.1 ± 1.03	18.76 ± 0.94	17.9 ± 0.76	Transcutaneous left auricular VNS plus rehabilitation training for 3 weeks	Sham VNS plus rehabilitation training for 3 weeks	0.5 mA, 20 Hz, lasting 30s per time of every 2 mintues, total of 30 mintues for 3 weeks	FMA-UE, WMFT, FIM	The number of serious adverse events related to therapy; BP; HR	No adverse events

ARAT = action research arm test, BDI = the Beck depression inventory, BP = blood pressure, FIM = Functional Independence Measurement, FMA-UE = Fugl-Meyer Assessment-Upper Extremity, HR = heart rate, MAL = motor activity log, MFAS = motor function assessment scale, RCT = randomized control trail, SIS = Stroke Impact Scale, SS-QOL = stroke specific quality of life, VNS = vagus nerve stimulation, WMFT = Wolf motor function test.

### 3.3 Study quality

**Figure 2:**
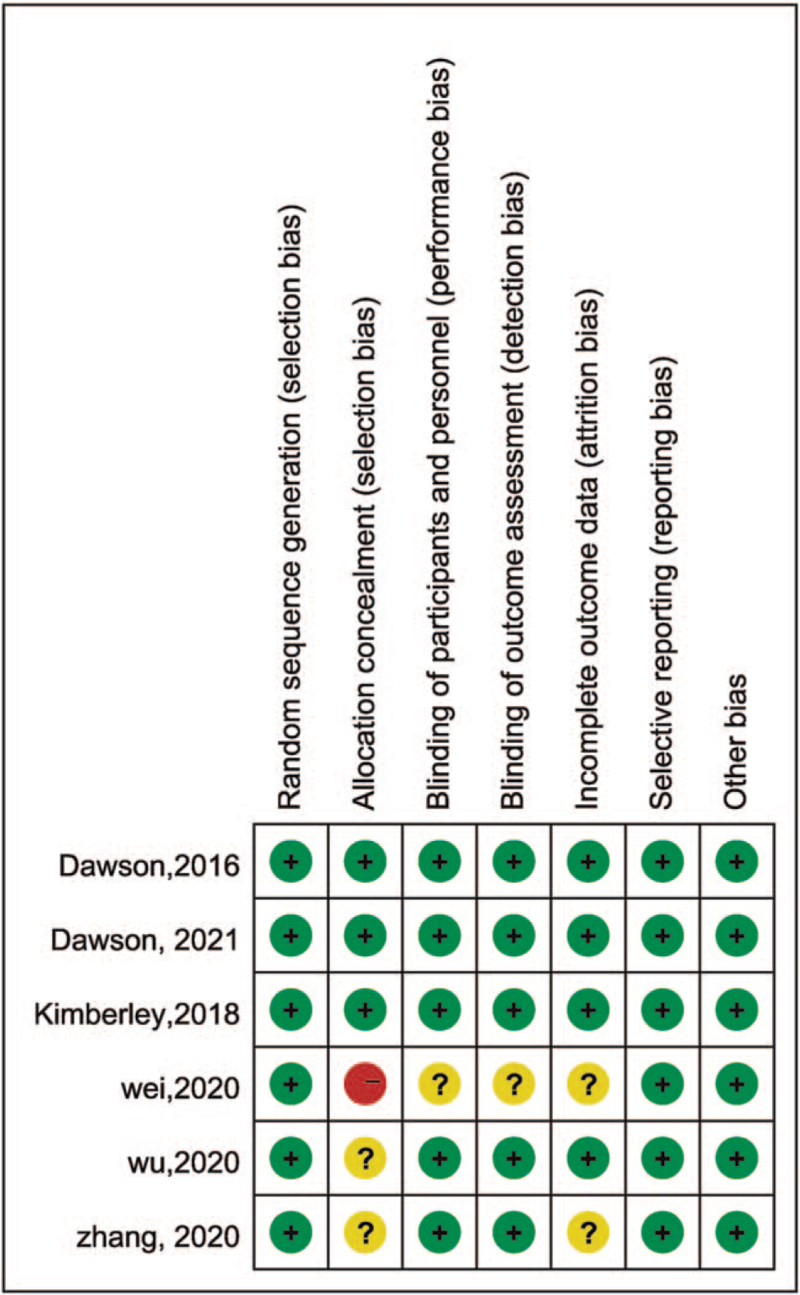
Risk of bias summary of included studies in this meta-analysis.

All included studies were RCTs. All of the included studies described the sequence generation method. Three studies illustrated the allocation concealment covering via email, phone call and/or an interactive voice response system. One study^[27]^ did not report the completeness of outcome data. The study of Wei^[26]^ did not describe the blinding and also had a high risk of bias on allocation concealment. Figure 2 describes the risk of bias in detail.

### 3.4 The primary outcomes

#### 3.4.1 Fugl-Meyer Assessment-Upper Extremity

**Figure 3:**
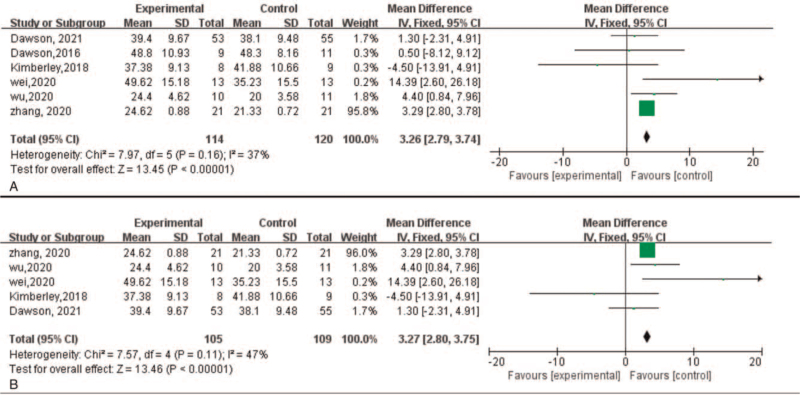
Forest plot of efficacy of VNS on motor function with FMA-UE. FMA-UE = Fugl-Meyer Assessment-Upper Extremity, VNS = vagus nerve stimulation.

FMA-UE primarily reflects the change of upper limb function. FMA-UE scores at the endpoint were available for all the selected studies. The simulated results were comparable with the control group, where VNS group has shown the higher change on FMA-UE scores (MD = 3.26, 95%CI [2.79, 3.74], *P* < .00001) with acceptable heterogeneity (χ^2^ = 7.97, *P* = .16, I^2^ = 37%) (Fig. 3A).

#### 3.4.2 The adverse events related to therapy or devices

**Figure 4:**
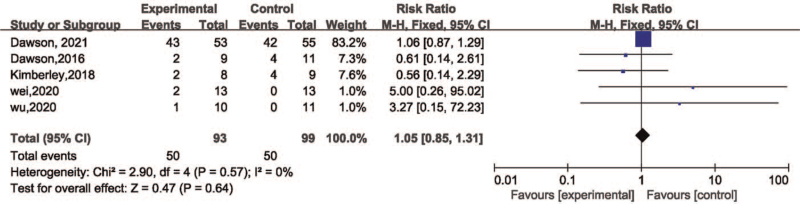
Forest plot for meta-analysis of safety of VNS on motor function. VNS = vagus nerve stimulation.

The adverse events associated with the therapy were reported in 5 studies^[17,20,24–26]^ as shown in Table 1. The simulated result revealed that the VNS was feasible and safe (risk ratio = 1.05, 95%CI [0.85, 1.31], *P* = .64) with no obvious heterogeneity in the obtained data (χ^2^ = 2.9, *P* = .57, I^2^ = 0%) (Fig. 4).

### 3.5 The secondary outcomes

**Figure 5:**
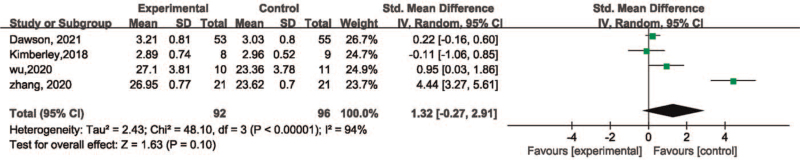
Forest plot for meta-analysis of efficacy of VNS on motor function with WMFT. VNS = vagus nerve stimulation, WMFT = Wolf motor function test.

**Figure 6:**
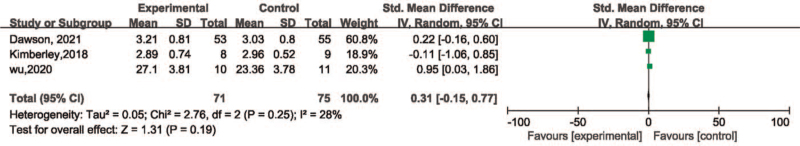
Forest plot for sensitivity analysis of efficacy of VNS on motor function with WMFT. VNS = vagus nerve stimulation, WMFT = Wolf motor function test.

Four studies^[17,24,25,27]^ reported WMFT, including 188 patients, however, significant heterogeneity was detected among the studies (χ^2^ = 48.10, *P* < .00001, I^2^ = 94%). Heterogeneity remained even after transferring the data into the random-effect model (Fig. 5) (χ^2^ = 48.10, *P* < .00001, I^2^ = 94%). Moreover, the simulated result revealed significant heterogeneity with no statistical difference among the groups (SMD = 1.32, 95%CI [–0.27, 2.91], *P* = .10). Each study was excluded orderly following the sensitivity analysis. After removing the study of Zhang et al, although the heterogeneity changed but no significant difference in simulated result (χ^2^ = 2.76, *P* = .25, I^2^ = 28%) (SMD = 0.31, 95%CI [–0.15, 0.77], *P* = .19) (Fig. 6).

**Figure 7:**
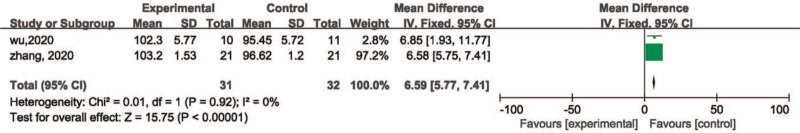
Forest plot for meta-analysis of efficacy of VNS on motor function with FIM. FIM = Functional Independence Measurement, VNS = vagus nerve stimulation.

Two studies^[25,27]^ including 63 patients reported FIM. The simulated results were comparable with control group, however VNS significantly improved limb motor function via FIM with no obvious heterogeneity (MD 6.59, 95%CI [5.77, 7.41], *P* < .00001) (χ^2^ = 0.01, *P* = .92, I^2^ = 0%) (Fig. 7).

### 3.6 Subgroup analysis

Subsequently, subgroup analysis was performed based on the intervention and duration of stroke to identify possible factors that might affect the efficacy of VNS on ischemic stroke.

**Figure 8:**
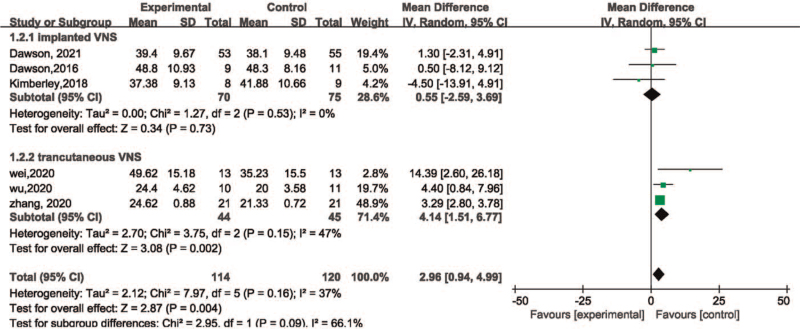
Forest plot for within intervention subgroup analysis of efficacy of VNS on motor function. VNS = vagus nerve stimulation.

In the subgroup of intervention, the group of transcutaneous VNS included 89 patients whereas the group of implanted VNS included 145 patients. It was observed that transcutaneous VNS (MD = 4.14, 95%CI [1.51, 6.77], *P* = .002) showed greater effect on patients after ischemic stroke than the implanted VNS (MD = 0.55, 95%CI [–2.59, 3.69], *P* = .73) (Fig. 8).

**Figure 9:**
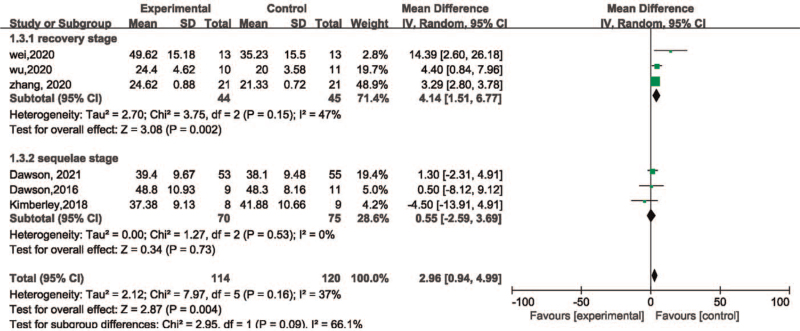
Forest plot for within stroke duration subgroup analysis of efficacy of VNS on motor function. VNS = vagus nerve stimulation.

The stroke durations of all the included patients were longer than 2 weeks. Hence, the value of 6 months was taken as the cutoff point, while dividing the durations into recovery and sequelae stages. The recovery stage group included 89 patients and the sequelae stage group included a total of 145 patients. The subgroup analysis of stroke duration indicated that the patients within recovery stage (MD = 4.14, 95%CI [1.51, 6.77], *P* = .002) demonstrated better enhancement in motor function in comparison with the sequelae stage (MD = 0.55, 95%CI [–2.59, 3.69], *P* = .73) (Fig. 9).

## 4 Discussion

Following the stroke, the recovery of upper limb impairment is relatively slower than that of the lower limb. Although a series of therapies have been applied to the clinical treatment, there is still a large number of patients suffering from upper limb impairment.^[28–30]^ Several RCTs are reporting VNS, as a promising tool for a feasible and effective gain of motor function after stroke, although there are only a few meta-analysis that have been done on this subject. There is a growing need for the simulated analysis underlying RCTs to ascertain the effect of VNS on poststroke motor impairment.

In the current meta-analysis, 6 studies including 234 patients were analyzed. We used FMA-UE, WMFT, FIM, and the number of adverse events to evaluate our simulated results. There was only a significant difference in the FMA-UE score between the groups, which further validates the use of VNS. Based on the pooled results, subgroup analysis on the intervention and duration of stroke were performed. The efficacies of both implanted and transcutaneous VNS on ischemic stroke have been proven in the pre-clinical and clinical trials, with emphasis on the importance on pairing VNS with rehabilitative exercises.^[2,18,31,32]^ It is speculated that transcutaneous VNS shares a similar pathway or mechanism with that of implanted VNS. The VNS causes stimulation mediated activation of brainstem vagi nuclei via afferent fibers of the vagus nerve, though there was no evidence to show whether the intensity of activated vagus nerve was maintained consistently between both the VNS.^[25,33]^ However, there is scarcity of studies which compares the efficacy of both VNS. The result of subgroup analysis revealed that the implanted VNS did not affect the motor function after ischemic stroke. Notably, the FMA-UE scores of reports by Dawson et al and Kimberley et al at baseline had a 5-point and 6-point difference between the studied groups, respectively. Therefore, this meta-analysis indicated that the transcutaneous VNS has superior benefits in improving the motor function in patients after ischemic stroke, whereas the implanted VNS might also be effective.

For implanted VNS groups, 1 study did not implant the device related VNS as the control group.^[20]^ Although the population weight of this one in included studies was small, to eliminate the effect of placebo, the other 5 studies were analyzed. The simulated result still showed the significant change on FMA-UE scores (MD = 3.27, 95%CI [2.80, 3.75], *P* < .00001) with acceptable heterogeneity (χ^2^ = 7.57, *P* = .11, I^2^ = 47%) (Fig. 3B) among groups, which seemed to identify the effectiveness of VNS.

Within-subgroup analysis of stroke duration suggested that compared with patients in the sequelae stage, those in the recovery stage had a significant change in motor function. A series of trials have identified that enhancement of neuroplasticity mediated by VNS paired with rehabilitation training, for the basis of motor function recovery poststroke.^[15,17,20,31]^ Interestingly, in comparison with chronic stroke, patients with sub-chronic stroke often demonstrate greater improvement in motor function.^[34]^ Similarly, taking the difference of FMA-UE at baseline in the study of Dawson et al and Kimberley et al into consideration, VNS can improve motor function in patients with sub-chronic stroke, and might also be effective for those with chronic stroke.

Based on subgroup analysis, the Chinese cohorts were given transcutaneous VNS during the recovery stage while the White cohorts treated by implanted VNS during the sequelae stage. In view of different religious beliefs, economics, sociology, and cultures, the acceptance of VNS varied among each race. Previous study showed the racial disparities in access to VNS devices.^[35]^ Therefore, ethnicity might be an influence factor for these outcome measures.

VNS also showed positive effects on WMFT and FIM. Based on the sensitivity analysis of WMFT, the study of Zhang et al was considered as the source of heterogeneity, due to the different stimulation parameters, unclear allocation concealment, and sample size.

There was no significant difference in safety between the studied groups. According to the data from 6 studies, only a few patients reported the occasional slight discomfort, whereas none of the severe events were reported associated with the device. Hence, VNS was deemed significantly safe for upper limb impairment after ischemic stroke.

In the current analysis, there was a great difference in the proportion of male and female although no significant differences among groups. Failing to obtain valid data, the different effects of VNS on sexuality could not be analyzed. Fortunately, there were studies reporting the sex differences in hemodynamic and autonomic regulation of cardiovascular systems both on animal and human trials.^[36,37]^ In terms of adverse effects of VNS, female subjects were more likely to express side effects than that of males, and this difference may originate from discrepancy in the sensitivity of certain nuclei following the cardiac branch pathway in female and male subjects.^[38,39]^ On the difference of the curative effects of VNS, female subjects also performed less effectiveness.^[40]^ These findings might be the basic evidence for future researches exploring the response of sexuality to VNS.

While the mechanisms of VNS are still unclear, it is speculated that it may be associated with the neuroprotection within the acute stage^[41–44]^ and enhancement of neuroplasticity during poststroke.^[14,45]^ The neuroprotection included: induction of neoangiogenesis to reduce infarct volume, alleviate neuron damage and enhance neurofunction.^[46]^ Suppression of inflammation via activating the cholinergic anti-inflammatory pathway.^[47]^ Adjustment in the level of malondialdehyde, glutathione, and superoxide dismutase in brain regions for suppressing the cellular responses to oxidative stress.^[48]^ As discussed earlier, VNS could also enhance synaptic plasticity via release of neurotransmitters.^[15]^ Furthermore, studies have reported that the VNS promoted the level of brain-derived neurotrophic factor, which in turn triggered nerve regeneration and enhances synaptic plasticity.^[49]^

The optimal parameters of VNS are explored to increase the degree of VNS-dependent neuroplasticity. Pruitt et al^[50]^ reported an inverted-U relationship between stimulation intensity with the motor function recovery, therefore suggesting the moderate-intensity VNS (0.8 mA) paired with rehabilitation for a significant yield of greater functional recovery than lower (0.4 mA) and higher stimulation intensity (1.6 mA), although the mechanism underlying this relationship was not defined. The same relationship was detected for the stimulation frequency, where the moderate-frequency (30 Hz) enhanced the cortical plasticity than the slower (7.5 Hz) and faster (120 Hz) pulse rate.^[51]^ Overall, the above studies elucidated the influence of different stimulation parameters on motor function recovery. Although, more studies are required to explore and validate the most optimal program.

There were some limitations in this meta-analysis. First, considering the number of included studies, the sample size of each study, the quality of studies, and simulated synthesis, the conclusions from simulated results must be interpreted with caution. Second, the dose parameters were varying for the included studies such as stimulation intensity, frequency, and training duration of VNS. At present, there is no standard recommendation for the parameters for using VNS,^[50]^ therefore, the efficacy of VNS may vary with the change in parameters. Lastly, it is worth noting that the patients enrolled in the included studies might not be the true representation of patients with upper limb impairment after ischemic stroke worldwide.

## 5 Conclusion

Overall, this meta-analysis demonstrated that the VNS is feasible and safe for patients with upper limb impairment after ischemic stroke. Poststroke, use of VNS showed an improvement in motor function in patients, and especially for those in the sub-chronic stage. Moreover, compared with the implanted VNS, transcutaneous VNS was more effective for patients after ischemic stroke. However, due to the above-mentioned limitations, future multicentric studies with larger sample RCTs are required to optimize the stimulation parameters and to identify the efficacy of VNS on motor function after stroke.

## Author contributions

**Conceptualization:** Yu-lei Xie, Shan Wang.

**Data curation:** Shan Wang.

**Formal analysis:** Xin Chen.

**Methodology:** Shan Wang.

**Project administration:** Qing Wu.

**Software:** Yu-lei Xie.

**Supervision:** Yu-lei Xie, Shan Wang.

**Validation:** Qing Wu.

**Visualization:** Yu-lei Xie.

**Writing – original draft:** Yu-lei Xie.

**Writing – review & editing:** Shan Wang.
